# Peripheral blood clinical laboratory variables associated with outcomes following combination nivolumab and ipilimumab immunotherapy in melanoma

**DOI:** 10.1002/cam4.1356

**Published:** 2018-02-22

**Authors:** Samuel Rosner, Erica Kwong, Alexander N. Shoushtari, Claire F. Friedman, Allison S. Betof, Mary Sue Brady, Daniel G. Coit, Margaret K. Callahan, Jedd D. Wolchok, Paul B. Chapman, Katherine S. Panageas, Michael A. Postow

**Affiliations:** ^1^ Department of Medicine Johns Hopkins Bayview Medical Center Baltimore Maryland; ^2^ City University of New York at Hunter College New York City New York; ^3^ Memorial Sloan Kettering Cancer Center New York City New York; ^4^ Weill Cornell Medical College New York City New York; ^5^ Ludwig Center for Cancer Immunotherapy New York City New York

**Keywords:** Biomarkers, immunotherapy, ipilimumab, nivolumab, PD‐1, pembrolizumab, prognosis

## Abstract

Both the combination of nivolumab + ipilimumab and single‐agent anti‐PD‐1 immunotherapy have demonstrated survival benefit for patients with advanced melanoma. As the combination has a high rate of serious side effects, further analyses in randomized trials of combination versus anti‐PD‐1 immunotherapy are needed to understand who benefits most from the combination. Clinical laboratory values that were routinely collected in randomized studies may provide information on the relative benefit of combination immunotherapy. To prioritize which clinical laboratory factors to ultimately explore in these randomized studies, we performed a single‐center, retrospective analysis of patients with advanced melanoma who received nivolumab + ipilimumab either as part of a clinical trial (*n* = 122) or commercial use (*n* = 87). Baseline routine laboratory values were correlated with overall survival (OS) and overall response rate (ORR). Kaplan–Meier estimation and Cox regression were performed. Median OS was 44.4 months, 95% CI (32.9, Not Reached). A total of 110 patients (53%) responded (CR/PR). Significant independent variables for favorable OS included the following: high relative eosinophils, high relative basophils, low absolute monocytes, low LDH, and a low neutrophil‐to‐lymphocyte ratio. These newly identified factors, along with those previously reported to be associated with anti‐PD‐1 monotherapy outcomes, should be studied in the randomized trials of nivolumab + ipilimumab versus anti‐PD‐1 monotherapies to determine whether they help define the patients who benefit most from the combination versus anti‐PD‐1 alone.

## Introduction

The combination of nivolumab + ipilimumab is highly effective in the treatment of patients with advanced melanoma 1, 2, 3 and is being explored in other malignancies. Due to a higher rate of serious initial side effects compared to single‐agent anti‐programmed death 1 (PD‐1) therapy, identifying which patients may benefit most from the combination is critical. Only analyses within randomized trials of combination immunotherapy versus anti‐PD‐1 monotherapy can truly identify which patients benefit most from the combination. Nonetheless, little is known about which factors to explore in these randomized trials. Specifically, to our knowledge, no prior studies have been published reporting routine clinical laboratory variables as possibly related to outcomes for nivolumab + ipilimumab combination immunotherapy.

Many potential biomarkers have been proposed for single‐agent ipilimumab and anti‐PD‐1 therapies. Most have involved immunologic aspects of the tumor microenvironment such as the expression of PD‐L1 4, 5, the presence of tumor‐infiltrating T cells 6, a high mutational load 7, 8, and various specific molecular signatures, including loss of phosphatase and tensin homolog (PTEN) 9. Nonetheless, results from biomarker analyses performed on tumors may be inherently limited due to immunologic and genetic heterogeneity between tumors within an individual patient 10 and may require on‐treatment immunologic assessment 11.

Basic peripheral blood laboratory variables obtained in routine clinical care prior to treatment initiation may also be important biomarkers in immunotherapy 12, 13. As these basic variables are routinely collected as part of standard care, these variables can be studied in large patient populations, including in randomized clinical trials. Ultimately, if validated in randomized trials, basic peripheral blood laboratory variables could be most easily applied to clinical practice.

We therefore evaluated widely available peripheral blood laboratory values and basic clinical characteristics in patients with advanced melanoma treated with the combination of nivolumab + ipilimumab to determine which variables are associated with objective response rate (ORR) and overall survival (OS). After defining favorable factors for combination immunotherapy, we were then interested in a separate, yet related hypothesis that the greatest difference in ORR and OS between combination immunotherapy and anti‐PD‐1 monotherapy exists among patients who are not expected to do well with anti‐PD‐1 monotherapy.

## Materials and Methods

We performed a retrospective study of 209 patients with unresectable stage III or IV melanoma treated with the combination of nivolumab + ipilimumab (*n* = 122 from phase I‐III clinical trials and the expanded access program; *n* = 87 from commercial use) at Memorial Sloan Kettering Cancer Center (MSKCC). This project was approved by the MSKCC Institutional Board Review. Baseline peripheral blood samples were obtained in the routine course of clinical care between 0 and 14 days before the first dose of combination immunotherapy.

Five basic laboratory parameters, readily available from standard of care laboratory testing, were analyzed: lymphocytes, eosinophils, monocytes, neutrophils, and basophils. These five variables were studied in relationship to clinical outcomes both as absolute (total number of cells in thousands per microliter) and relative (percentage of total white blood cells) values. The term “relative” was selected for consistency with prior literature on this topic 13. Further, the derived absolute neutrophil‐to‐lymphocyte ratio (NLR) was studied as it has previously been associated with outcomes for patients with melanoma receiving immunotherapy 14, 15, 16. LDH and M‐stage, well‐known prognostic variables, were additionally analyzed. LDH was dichotomized using the institutional upper limit of normal of ≤ or >246 units per liter (U/L), and M‐stage was categorized as per American Joint Committee on Cancer Staging 7th Edition (M1a, M1b, M1c). M0 refers to patients who did not have metastatic disease but had unresectable stage III disease.

To investigate continuous data as categorical groups (high vs. low), optimally selected cut‐points for laboratory values were estimated based on maximally selected log‐rank statistics and significance was assessed with Lausen and Schumacher's 17 *P*‐value approximation. *P* values <0.10 were considered statistically significant.

Follow‐up time was defined as the time from the first dose of treatment to the date of last known contact or death. Survival probabilities and median OS with 95% confidence intervals (CI) were estimated according to the Kaplan–Meier method and compared using log‐rank tests. Multivariate analysis results from Cox models are described by hazard ratios (HRs) with 95% CIs, and *P* values are based on the Wald test.

All responses were investigator assessed as per RECIST 1.1 criteria 18 except for patients in the phase I trial of nivolumab + ipilimumab who had responses assessed by the modified World Health Organization Criteria (mWHO). Responses for patients off protocol were investigator assessed. Patient responses were categorized as either complete response (CR), partial response (PR), stable disease (SD), or progressive disease (PD).

To descriptively report overall survival following the nivolumab + ipilimumab combination by the number of adverse laboratory variables present, we pooled patients with 0–1, 2–3, and 4–5 adverse values. Five adverse values were selected which were significant in multivariate analysis. Patients who had any missing values were excluded.

Additionally, to descriptively report how patients expected to do poorly with anti‐PD‐1 monotherapy did with combination immunotherapy in our dataset, we selected four factors previously reported to be independently associated with inferior anti‐PD‐1 monotherapy ORR and OS: nonlung visceral metastases versus any other metastatic site; LDH ratio >2.5 versus ≤2.5 times the upper limit of normal; relative lymphocytes <17.5 versus ≥17.5; and relative eosinophils <1.5 versus ≥1.5 13. We then descriptively reported ORR and OS among patients treated with combination immunotherapy in our dataset that had 0, 1, 2, 3, or 4 of these previously described unfavorable factors for PD‐1 monotherapy outcomes.

## Results

Patient (*n* = 209) demographics are shown in Table 1. The majority of patients (74.6%) were treatment‐naïve. Fifty‐eight (27.8%) died during follow‐up. For patients alive at last follow‐up (*n* = 151), the median duration of follow‐up was 13.1 months. Estimated median OS was 44.4 months, 95% CI [32.86‐NR] (Fig. S1). The overall response rate was 52.6% (11% CR and 41.6% PR); 18.6% had SD, and 24.4% had PD. Patients who died before obtaining a postbaseline scan were considered to have PD; 4.3% had unknown response assessment (no postbaseline scan and no documented death).

**Table 1:** Patient Demographics (*n* = 209)

	*N* (%)
Age at treatment start, years	
Median (range)	60.5 (22.0–86.4)
Gender	
Male	124 (59.3)
Female	85 (40.7)
M‐stage	
M0	40 (19.1)
M1A	18 (8.6)
M1B	34 (16.3)
M1C	117 (56)
Prior systemic treatment	
Yes	53 (25.3)
No	156 (74.6)
Prior treatment, type	
None	156 (74.6)
Chemotherapy	9 (4.3)
PD1	18 (8.6)
Ipilimumab	12 (5.7)
BRAF/MEK Inhibitors	23 (11)
Other	16 (7.6)
Prior treatment, number of lines	
Median (range) (*N* = 209)	0 (0–7)
Last follow‐up status	
Dead	58 (27.8)
Alive	151 (72.2)

As continuous variables, seven laboratory parameters were significantly associated with OS. Higher relative lymphocytes, relative eosinophils, and relative basophils were significantly correlated with improved OS. Higher absolute monocytes, absolute neutrophils, and relative neutrophils were significantly correlated with worse OS. Increasing NLR was also significantly associated with worse OS. (Table 2) No individual variables correlated with objective response (CR/PR vs. SD/PD).

**Table 2:** Continuous variables examined for association with overall survival

Factors of Interest	Number of Patients	Number of Events	HR 95% CI	*P*‐value
Absolute lymphocytes	209	58	0.85 (.57, 1.26)	0.41
Relative lymphocytes	209	58	0.93 (.90, .97)	<0.001^a^
Absolute eosinophils	209	58	0.19 (.02, 2.39)	0.20
Relative eosinophils	209	58	0.73 (.59, .90)	0.004^a^
Absolute monocytes	209	58	9.31 (4.07, 21.3)	<0.0001^a^
Relative monocytes	209	58	1.03 (.99, 1.07)	0.21
Absolute neutrophils	209	58	1.20 (1.13, 1.28)	<0.0001^a^
Relative neutrophils	209	58	1.06 (1.02, 1.09)	<0.001^a^
Absolute basophils	209	58	6.64 (.13, 342.8)	0.35
Relative basophils	209	58	0.34 (.15, .79)	0.01^a^
Neutrophil‐to‐lymphocyte ratio	209	58	1.19 (1.12, 1.28)	<0.0001^a^

a indicates significant *P*‐value.

**Figure 1:**
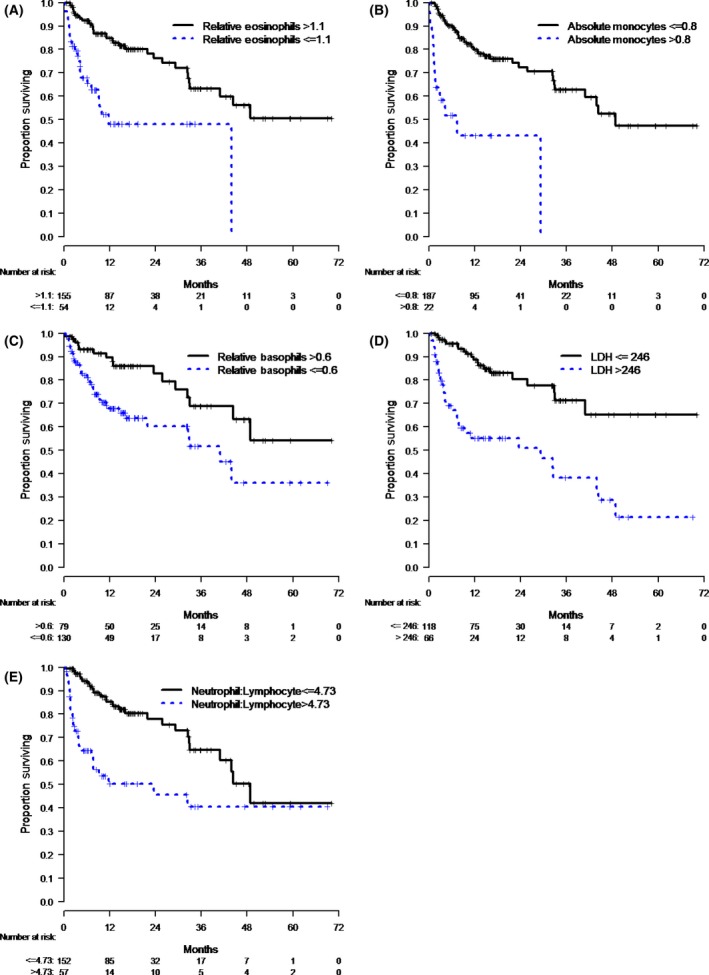
(A–E): Categorical variables which were significantly associated with OS. (A) Patients with relative eosinophils ≤1.1 had worse OS versus those with relative eosinophils >1.1 (HR 3.48, *P* < 0.0001). (B) Patients with absolute monocytes >0.8 had worse OS versus those with absolute monocytes ≤0.8 (HR 5.56, *P* < 0.000). (C) Patients with relative basophils ≤0.6 had worse OS versus those with relative basophils >0.6 (HR 2.33, *P* = 0.0). (D) Patients with LDH >246 U/L had worse OS versus those with LDH ≤246 U/L (HR 3.83; *P* < 0.0001). (E) Patients with NLR >4.73 had worse OS versus those with NLR ≤4.73 (HR 2.95, *P* < 0.0001).

To facilitate possible ultimate clinical utility and for data visualization, the seven continuous variables that were significantly associated with OS were then dichotomized into high versus low groups based on a cut‐point analysis. LDH and M‐stage were additionally considered as categorical variables as described in Methods. Among these categorical variables, five variables were significantly associated with OS (Table 3, Fig. 1): relative eosinophils ≤1.1 versus >1.1 (HR 3.48, *P* < 0.0001), absolute monocytes >0.8 versus ≤0.8 (HR 5.56, *P* < 0.0001), relative basophils ≤0.6 vs. >0.6 (HR 2.33, *P* = 0.005), LDH >246 vs. ≤246 U/L (HR 3.83; *P* < 0.0001), and NLR >4.73 vs. ≤4.73 (HR 2.95, *P* < 0.0001).

**Table 3:** Univariate analysis of factors associated with overall survival

Factor of interest	Hazard Ratio (95% CI)	*P*‐value
Relative eosinophils (≤1.1 vs. >1.1)	3.48 (2.02, 6.01)	<0.0001
Absolute monocytes (>0.8 vs. ≤0.8)	5.56 (2.88, 10.74)	<0.0001
Relative basophils (≤0.6 vs. >0.6)	2.33 (1.30, 4.19)	0.005
LDH (>246 vs. ≤246)	3.83 (2.19, 6.69)	<0.0001
NLR (>4.73 vs. ≤4.73)	2.95 (1.75, 4.97)	<0.0001

All five of these categorical variables retained significance in a multivariate model. Low relative eosinophils (HR 2.38, *P* = 0.007) and low relative basophils (HR 1.85, *P* = 0.08) were found to be independently associated with worse OS. High levels of absolute monocytes (HR 2.75, *P* = 0.01), LDH (HR 3.71, *P* < 0.0001), and the NLR (HR 1.95, *P* = 0.02) were also independently associated with worse OS (Table 4).

**Table 4:** Multivariate analysis of factors associated with overall survival

Factor of interest	Hazard Ratio (95% CI)	*P*‐value
Relative eosinophils (≤1.1 vs. >1.1)	2.38 (1.27, 4.46)	0.007
Absolute monocytes (>0.8 vs. ≤0.8)	2.75 (1.30, 5.80)	0.01
Relative basophils (≤0.6 vs. >0.6)	1.85 (0.94, 3.66)	0.08
LDH (>246 vs. ≤246)	3.71 (2.08, 6.61)	<0.0001
NLR (>4.73 vs. ≤4.73)	1.95 (1.11, 3.43)	0.02

**Figure 2:**
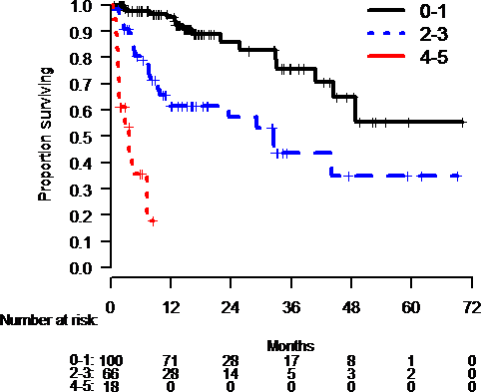
Overall survival of patients in our dataset treated with nivolumab + ipilimumab combination based upon the number of unfavorable variables for nivolumab + ipilimumab defined in this manuscript. As the number of unfavorable factors for combination immunotherapy outcomes increases, the OS of patients treated with the combination in our dataset generally decreases.

Using these five variables which were significant in a multivariate model, we then examined patients with 0, 1, 2, 3, 4, and 5 adverse factors. Due to low numbers within each group, we pooled patients with 0–1, 2–3, and 4–5 adverse factors (Fig. 2). Descriptively, there was an inverse relationship between the number of unfavorable variables present and overall survival following nivolumab + ipilimumab.

**Figure 3:**
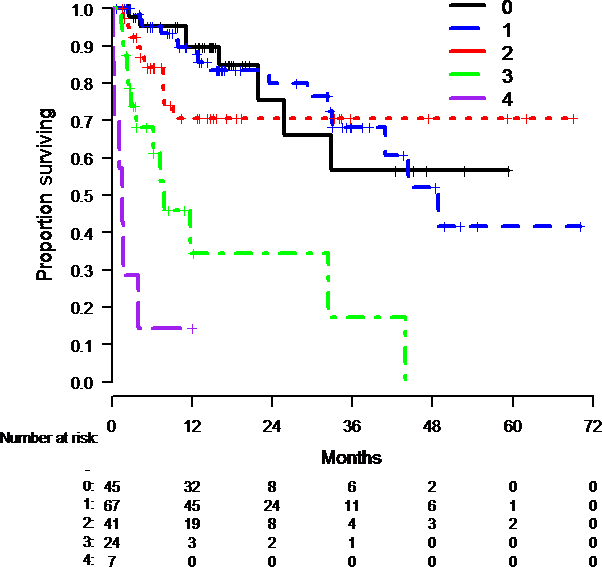
Overall survival of patients in our dataset treated with nivolumab + ipilimumab combination based upon the number of unfavorable variables for anti‐PD‐1 monotherapy (as described in 13). As the number of unfavorable factors for anti‐PD‐1 outcomes increased, the OS of patients treated with the combination in our dataset generally decreased.

Next, we wanted to preliminarily describe how patients expected to have poorer outcomes with anti‐PD‐1 monotherapy did with combination immunotherapy in our dataset. As described in Methods, we looked at the response rates and OS among patients in our dataset with 0, 1, 2, 3, or 4 previously described poor prognostic variables for pembrolizumab monotherapy 13. There was an inverse relationship between the number of unfavorable factors for anti‐PD‐1 monotherapy and response rate to combination immunotherapy in our dataset. The same was generally true for median OS. (Table 5, Fig. 3).

**Table 5:** Response rate and overall survival of patients treated with nivolumab + ipilimumab combination immunotherapy based upon the number of unfavorable variables for anti‐PD‐1 monotherapy (as described in 13)

Number of Unfavorable Anti‐PD‐1 Variables	Response Rate with Combination^a^ 95% CI	Median Overall Survival with Combination (months) 95% CI
0 (*n* = 42)	69.1% (52.9%–82.4%)	Not Reached (22.0, Not Estimable)
1 (*n* = 64)	54.7% (41.8%–67.2%)	48.9 (33.0, Not Reached)
2 (*n* = 40)	55.0% (38.5%–70.7%)	Not Reached (Not Estimable)
3 (*n* = 23)	43.5% (23.2%–65.5%)	7.9 (3.7, 32.4)
4 (*n* = 6)	16.7% (0.4%–64.1%)	1.5 (0.3, 3.9)

a Calculated by total # of responses/total # of patients with this number of anti‐PD‐1 unfavorable variables.

## Discussion

In this study, we define five routine clinical peripheral blood laboratory values (relative eosinophils, relative basophils, absolute monocytes, LDH, and NLR) that are independently associated with OS in patients treated with the combination of nivolumab + ipilimumab. As several of these factors such as LDH are established prognostic markers, we are unable to determine whether these factors are predictive of outcome or only prognostic. Several of the unfavorable variables (monocytes, LDH, and NLR) as well as a favorable variable (eosinophils) are consistent with prior studies of ipilimumab or anti‐PD‐1 as single agents 12, 13, 14, 19, 20, 21, 22. It is therefore possible that these cell populations have a mechanistic role in immunotherapy outcomes.

Eosinophils have been shown to exert favorable effects on antitumor immunity in preclinical models. Eosinophils may be important mediators in recruiting T cells to the tumor microenvironment 23 which may aid immunologic tumor control as tumors heavily infiltrated with T cells are believed to be more responsive to anti‐PD‐1‐based immunotherapy 6. Other studies suggest eosinophils play a direct role in limiting the process of carcinogenesis and can kill tumor cells directly 24.

In addition to eosinophils, we found that patients with a high proportion of basophils had better overall survival. To our knowledge, basophils have not been previously described as a relevant cell population in patients receiving immune checkpoint inhibitors. Why some cells of the myeloid lineage (eosinophils and basophils) but not all (monocytes) were associated with favorable effects remains unclear. Nonetheless, our finding that monocytes were negatively associated with outcomes is consistent with other studies 12, 25. Monocytes may exert unfavorable effects on antitumor immunity via many mechanisms, including giving rise to immunosuppressive tissue‐resident, M2‐macrophages 26. Many strategies to therapeutically target immunosuppressive myeloid populations are being tested 27, making our findings relevant to this area of additional research.

Our study has several limitations. First, our study cohort was from a single institution, raising the risk for regional, site‐specific, or physician treatment bias. Nonetheless, the response rate in our study is generally similar to that seen in larger phase studies of nivolumab + ipilimumab combination therapy 2, 3. Although the median OS has not yet been reached in prior studies of combination nivolumab + ipilimumab in treatment‐naïve patients, our median OS was reached at 44.4 months. This may have been the case as we included patients who received treatment off protocol; approximately one‐quarter of patients also had prior treatment before beginning combination immunotherapy. Our study also did not examine whether these variables were related to side effects. As prior studies have implicated variables such as eosinophilia with toxicity 28, this analysis remains of interest for future investigations in larger populations where associations with these variables and specific toxicities may be able to be determined. Further, the fact that some variables were only associated with outcomes when considered as a relative percent versus absolute number remains unclear. This suggests, however, that the balance among several cell populations may be more important than the total number in some contexts. Finally, we found significant associations between our investigated factors and OS but not ORR. Between these two endpoints, we feel that OS is more important as the critical question remains whether there are any patient populations that derive OS benefits from combination immunotherapy versus anti‐PD‐1 monotherapy. In contrast to OS, most prior analyses of randomized trials have already shown that the ORR is generally greater for combination immunotherapy versus PD‐1 monotherapy across subgroups [3]. Nonetheless, the association of these variables with OS may simply indicate they are prognostic, rather than predictive.

Only a randomized trial can answer the question of who obtains the greatest benefit from the nivolumab + ipilimumab combination compared to single‐agent anti‐PD‐1. Nonetheless, based on these data and previously published data, it appears that patients who have no unfavorable anti‐PD‐1 variables (as described in 13) have excellent response rates and OS with either anti‐PD‐1 monotherapy 13 or nivolumab + ipilimumab combination therapy as shown in our dataset.

However, patients with 2 (ORR 24.3% and median OS of 4.2 months with anti‐PD‐1 monotherapy in 13 vs. ORR 55% and median OS of not reached with combination in our dataset) or 3 unfavorable variables for anti‐PD‐1 monotherapy (ORR 7.7% and median OS 1.4 months with anti‐PD‐1 monotherapy in 13 vs. ORR 43.5% and median OS of 7.9 months with combination immunotherapy in our dataset) may be the ones who receive the greatest benefit from the combination. Nevertheless, these preliminary comparisons between nonrandomized retrospective cohorts are not ready for clinical application. Ongoing randomized studies such as the Checkmate 067 study of nivolumab + ipilimumab or nivolumab monotherapy versus ipilimumab, with recently reported 3‐year overall survival results [29], provide an opportunity to more formally answer this question.

## Conflict of Interests

Rosner, Kwong, Friedman, Betof, Brady, Coit, Panageas: n/a. Shoushtari: Research support: BMS; Advisory board: Castle Biosciences, Immunocore, and Vaccinex. Callahan: Research support: BMS. Wolchok: Stock or other ownership in Potenza Pharmaceuticals and Vesuvius Pharmaceuticals; Honorarium from Regeneron; advisory/consulting for BMS, Merck, MedImmune, ZIOPHARM Oncology, Polynoma, Polaris, Jounce Therapeutics, Genentech, FStar, BeiGene, Advaxis, Sellas Life Sciences, Lilly, Potenza Therapeutics, Tizona Therapeutics Inc, Amgen, AstraZeneca, and Chugai Pharma; institutional research support from BMS; travel expenses from BMS, Chugai Pharma, Roche, Janssen, Kadmon, and Regeneron. Chapman: Honoraria: BMS, GlaxoSmithKline, Genentech/Roche, Provectus, Momenta Pharmaceuticals, and Daiichi Sankyo; Advisory board: BMS, GlaxoSmithKline, Genentech/Roche, Daiichi Sankyo, Provectus, and Momenta Pharmaceuticals; Research support: BMS, Genentech/Roche, GlaxoSmithKline, and Pfizer; Travel expenses: BMS. Postow: Research support: BMS; Advisory board: BMS, Merck, Incyte, NewLink Genetics, Array BioPharma, and Novartis; Honoraria: Merck and BMS.

## Supporting information


**Figure S1**. Overall Survival of the entire patient cohort (*n* = 209).
