# Supplementary material for: Peripheral blood clinical laboratory variables associated with outcomes following combination nivolumab and ipilimumab immunotherapy in melanoma
Source: Cancer Med. 2018 Feb 22;7(3):690–7. doi: 10.1002/cam4.1356 (PMC5852343; doi:10.1002/cam4.1356)
Supplement: Supplementary file 1 — Figure S1. Overall Survival of the entire patient cohort (n = 209). [file CAM4-7-690-s001.docx]

**Supplementary Figure S1: Overall Survival of the entire patient cohort (n=209)**

Estimated median overall survival was 44.4 months, 95% CI [32.86-NR] **
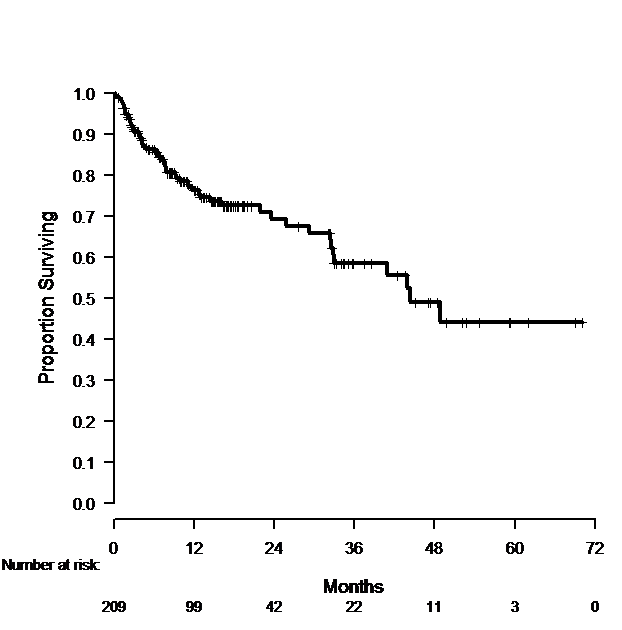
**
