# Supplementary figures and images for: Peripheral blood clinical laboratory variables associated with outcomes following combination nivolumab and ipilimumab immunotherapy in melanoma
Source: Cancer Med. 2018 Feb 22;7(3):690–7. doi: 10.1002/cam4.1356 (PMC5852343; doi:10.1002/cam4.1356)

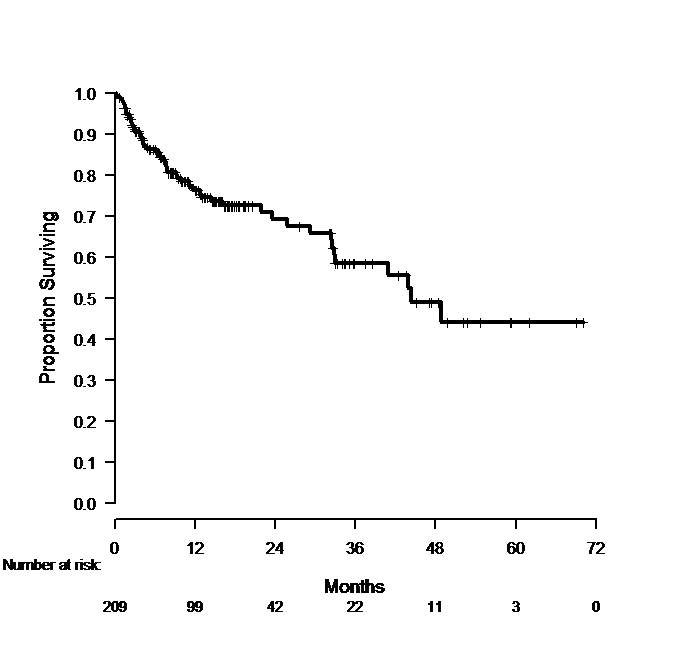

Supplement: Supplementary file 2 [file CAM4-7-690-s002.tif]
